# Bioactive Dairy-Fermented Products and Phenolic Compounds: Together or Apart

**DOI:** 10.3390/molecules28248081

**Published:** 2023-12-14

**Authors:** Barbara Wróblewska, Aleksandra Kuliga, Kinga Wnorowska

**Affiliations:** Institute of Animal Reproduction and Food Research, Polish Academy of Science, 10-748 Olsztyn, Poland; a.kuliga@pan.olsztyn.pl (A.K.); aagnikowaa@gmail.com (K.W.)

**Keywords:** milk and its fermented products, phenolic compounds, interactions, combined products, gastrointestinal tract, docking

## Abstract

Fermented dairy products (e.g., yogurt, kefir, and buttermilk) are significant in the dairy industry. They are less immunoreactive than the raw materials from which they are derived. The attractiveness of these products is based on their bioactivity and properties that induce immune or anti-inflammatory processes. In the search for new solutions, plant raw materials with beneficial effects have been combined to multiply their effects or obtain new properties. Polyphenols (e.g., flavonoids, phenolic acids, lignans, and stilbenes) are present in fruit and vegetables, but also in coffee, tea, or wine. They reduce the risk of chronic diseases, such as cancer, diabetes, or inflammation. Hence, it is becoming valuable to combine dairy proteins with polyphenols, of which epigallocatechin-3-gallate (EGCG) and chlorogenic acid (CGA) show a particular predisposition to bind to milk proteins (e.g., α-lactalbumin β-lactoglobulin, αs1-casein, and κ-casein). Reducing the allergenicity of milk proteins by combining them with polyphenols is an essential issue. As potential ‘metabolic prebiotics’, they also contribute to stimulating the growth of beneficial bacteria and inhibiting pathogenic bacteria in the human gastrointestinal tract. In silico methods, mainly docking, assess the new structures of conjugates and the consequences of the interactions that are formed between proteins and polyphenols, as well as to predict their action in the body.

## 1. Introduction

The development of the dairy market and the need to satisfy the dietary requirements of different consumer groups, including vulnerable ones (e.g., children, old, allergic, pregnant women, and people with gastrointestinal diseases), are triggers for designing new, attractive food products. Recently, fermented dairy products (e.g., yogurt, kefir, buttermilk, and others) have been perceived as showing beneficial health effects [1,2,3,4]. The literature data have been proven to emphatically reduce the incidence of health problems, including obesity, metabolic disorders, and cardiovascular diseases [5,6,7]. Enrichment of milk-fermented products with fruit, juice, pomace, or extracts rich in plant bioactive compounds is a promising direction to serve consumers with new attractive products with extended pro-health value [8,9,10,11]. At the same time, they contribute to the management of valuable by-products from side streams of fruits, vegetables, and the dairy industry.

The basic raw material of the dairy industry’s side streams is whey. Subjecting it to lactic fermentation allows beverages rich in bioactive peptides. Whey fermented with yogurt starters supplemented with *Lactobacillus plantarum* W42 and *Bifidobacterium animalis* ssp. *lactis* Bi30 had tolerogenic potential [12]. The results indicated an increase in total IgA and IgG, while it decreased the levels of total IgE analyzed in the blood serum of the mice. Whey altered the Th1/Th2 balance towards a Th1 response, showing an immunomodulatory effect. The bacteria used for fermentation significantly reduced levels of pro-inflammatory cytokines. Whey beverages are rich in bioactive peptides, with antimicrobial, antihypertensive, antithrombotic, antioxidant, mineral-binding, and antidiabetic properties [13,14]. Another valuable dairy raw material due to its high bioavailability, safety, and antimicrobial properties is colostrum [15]. Colostrum bioactive peptides were released with the utilization of fermentation by *Lactobacillus rhamnosus* C25, *Lactobacillus rhamnosus* C6, and *Lactobacillus casei* NCDC17 [16]. It is a rich source of immunoglobulins, fatty acids, and conjugated linoleic acid [17]. Colostrum has been successfully used as an additive in yogurts, resulting in products with good sensory acceptance [18,19]. The exploitation of the beneficial characteristics of raw materials and dairy products has led to an interest in combined dairy–plant products, with particular attention paid to the presence and role of polyphenols.

Polyphenols are secondary plant metabolites characterized by a broad spectrum of biological activities, mainly anti-inflammatory, immunomodulatory, antimicrobial, hepatoprotective, and gastroprotective. The mechanism of action of polyphenols in the inflammatory process is related to the control and inhibition of pro-inflammatory cytokine networks, such as IL-1β, IL-6, IL-8, and TNF-α, and enzymes involved in arachidonic acid metabolism. In addition, polyphenols exhibit anti-inflammatory activity at multiple levels through the inhibition of NF-κB, MAPK, and iNOS, along with the regulation of growth factors [20]. Comparing the prooxidant activity of (−)-epigallocatechin-3-gallate (EGCG) and chlorogenic acid (CGA) in vitro and in vivo (doses of 55 mg/kg EGCG and 42.5 mg/kg CGA) showed that EGCG exhibited strong prooxidant activity, whereas CGA showed none. CGA, together with a low dose of EGCG, synergistically activated hepatic AMPK and increased hepatic Nrf2-related proteins without causing toxicity in mice. Thus, it was demonstrated that a combination of polyphenols with different antioxidant values can be beneficial and cause no side effects [21]. Dietary polyphenols present in the human gastrointestinal tract can act as both antioxidants and prooxidants. In the intestinal environment, at the right pH, they can exhibit activity against pathogenic bacteria. Fermentation of protein–polyphenol mixtures can have a significant impact on maintaining homeostasis in the human gastrointestinal tract by releasing bioactive peptides and metabolites of polyphenols and altering their profile [22]. Polyphenols have a significantly high binding affinity to proteins and form complexes via interactions with active moieties in protein molecules [23]. This changes the structure, physical and chemical properties, and functions of proteins. The type of interaction (covalent or non-covalent) between components affects the final form of the resulting protein–polypeptide conjugates and their properties.

The indication of the interaction of bioactive plant phenolic components with high-value immunomodulatory milk protein components is still under debate and needs a detailed analysis. Due to technological processes, these individual components undergo changes, transforming into modified structures of adducts [24]. Further transformation of the food composition process takes place during consumption as a result of the digestion of the food complex. Gastrointestinal conditions (e.g., influence of enzymes, change in pH, the impact of gut microbiota, etc.) cause the hydrolysis of food compounds and direct enzymatic degradation in immunocompetent cells [25]. As a result, the cells of the immune system can recognize the antigen and respond with a specific immune reaction by producing antibodies and arranging the cytokine profile in a signaling path [26]. Scientific methods offer simulated digestion and in silico docking to predict potential enzymatic changes with nutrient compounds in the body and mimic the released generation of peptides from T cells [27,28].

Most of the available scientific data focuses on polyphenolic compounds commonly found in e.g., coffee, green tea such as epigallocatechin-3-gallate (EGCG) and chlorogenic acid [21] and dairy-derived proteins [29,30]. One important goal of such a combination is to reduce the allergenicity of milk proteins, which is an important issue in view of the continuing need to develop targeted nutrients for allergy sufferers [31]. It has been proven that protein–polyphenol conjugates inhibit the recognition of the allergen by previously produced specific antibodies due to the saturation of developed protein structures with molecules of metabolites of polyphenols [32].

The impact of food ingredients on organisms is a complex process, and working out an indication of the immunomodulatory potential of complex foods should be crucial before involving new products on the market.

This article presents current knowledge on dairy-fermented products, their biological activities, and fruit phenolic compounds. The authors point out the consequence of the passage of valuable health-promoting compounds through the digestive system by considering the problem of combining dairy proteins with polyphenols into adducts. The purpose of this article was to consider the benefits to the body of individual bioactive molecules and their activity. The optimal ratios of milk proteins to polyphenols to maximize bioavailability and immunoreactivity are still under debate.

## 2. Immunoreactivity of Dairy-Fermented Products

The world trend that has taken shape in recent years has been the issue of replacing milk with fermented products with lowering bio-converted milk proteins’ immunoreactivity, which has proved beneficial to people with allergies to cow’s milk proteins [33]. Their functional and nutritional potential was shown in the literature [34,35,36]. Immune properties are determined in terms of increased food tolerogenic by cow milk-allergic patients. The impact on the organism is also determined in terms of obtaining increased immune tolerogenicity by cow milk-allergic patients. Food allergies are one of the recognized diseases of civilization, and their emergence is still increasing. Food allergies affect 5–8% of children and adolescents, and about 1–3% of adults, mainly in highly developed countries. Cow’s milk, in addition to its nutritional and health-promoting properties, also contains proteins with allergenic potential [37]. Two main groups of proteins dominate the composition of milk: caseins (αs1-, αs2-, and κ-caseins) and whey proteins, mainly β-lactoglobulin (both strong allergens), α-lactalbumin, bovine serum albumin, and lactoferrin. Caseins are characterized by their loose, flexible structure [38], which are present in milk in the form of self-assembled particles known as “casein micelles” [39]. On the other hand, whey proteins are a group of globular proteins with a well-defined α-helix structure [38,40]. Research to reduce the allergenicity and immunoreactivity of milk proteins has been extensively conducted in the fields of thermal processes, enzymatic hydrolysis, bioconversion, and high pressure [41].

A number of fermented milk beverages are generally available, and their properties have been confirmed and described in the scientific literature. The most popular in the world, and a well-known fermented milk product, is yogurt. Traditionally, it is made with cow’s milk fermented with two strains of bacteria from the genera *Lactobacillus delbrueckii* ssp. *bulgaricus* and *Streptococcus thermophilus* [42]. Its health-promoting properties and beneficial effects on the digestive system have been described in the literature data [43,44,45]. The selection of lactic acid bacterial (LAB) strains to the fermentation dairy matrix is the key to achieving a reduction in product immunoreactivity. It was stated that the origin of the strains from one genus has not had the same properties, and their metabolic properties are more variable than initially thought. Dairy products manufactured with the use of lactobacilli starter cultures have different sensory properties, mainly texture, acidity, and flavor [46]. However, there are examples of enriching yogurts with other potentially beneficial bacteria, e.g., *Lactobacillus acidophilus* or *Bifidobacterium*. *Bifidobacterium* plays an important role during the maturation of the digestive system from birth to childhood. Experimenting with the use of Balb/c mice allows one to observe lower levels of the intestinal pro-inflammatory cytokines TNF-α, IFN-γ, and IL-2 and increased production of the anti-inflammatory cytokines IL-4 and IL-10. The authors showed the modulation of the TLR4/NF-κB signaling pathways to alleviate intestinal mucosal immune damage due to *Bifidobacterium longum*, Bi-10 [47]. During the study, the selected bacterial strains (*Streptococcus salivarius* ssp. *thermophilus* TKM3, *Streptococcus thermophilus* MK-10, *Streptococcus thermophilus* 2K, *Lactobacillus delbrueckii* ssp. *bulgaricus* DB3, *Lactobacillus bulgaricus* 151, *Lactobacillus bulgaricus* BK, *Bifidobacterium animalis* ssp. *lactis* Bi30, *Bifidobacterium lactis* J38, *Lactobacillus plantarum* W42, and *Lactobacillus plantarum* IB) originated from the collection of the Institute of Animal Reproduction and Food Research, Polish Academy of Science (IAR&FR PAS, Olsztyn, Poland), were used to reduce the immunoreactivity of milk. The yogurt strains were matched in pairs to form starter cultures, displaying synergistic effects to reduce the immunoreactivity of the new drink and stimulate the mice to restore their immune balance from Th2 into Th1. The most beneficial results were obtained with fermented products produced with a set of bacteria combined with *Streptococcus thermophilus* 2K and *Lactobacillus bulgaricus* BK strains and *Lactobacillus plantarum* W42 and *Bifidobacterium lactis* Bi30 strains. During an interventional experiment with Balb/c mice, the secretion of regulatory cytokines, i.e., IL-10 and TGF-β, and IgA increased, while the levels of IL-4, IgE, and anti-(α-CN + β-LG) IgG1 decreased. The authors concluded that fermented drinks with reduced antigenic potential present the allergoids that could be used in the immunotherapy of IgE-mediated cow’s milk allergy (CMA) [3]. Process temperature, storage conditions, and additives play a crucial role in the quality of yogurt. Starter culture YO-MIX495 and the addition of microbial transglutaminase (m-TG) both at temperatures of 37 °C and 42 °C reduced the immunoreactivity of yogurt except for α-and β-casein (fermented at 42 °C) in storage samples (28 days). The sensory analysis demonstrated that the addition of m-TG did not reduce the sensory quality of the yogurt obtained with YO-MIX495 [48].

The pandemic risk caused the interest in bacterial strains as being useful in the fight against influenza H1N1 and SARS-CoV-2 infection. Studies have been carried out through in vivo tests using influenza H1N1-infected C57BL/6 mice and SARS-CoV-2-infected Syrian golden hamsters. Yogurt caused an increase in the survival rate, body weight, and IFN-γ, IgG1, and IL-10 levels against viral infection and a decrease in the inflammatory cytokines TNF-α and IL-6 in influenza H1N1-infected (C57BL/6) mice. Experiments with SARS-CoV-2-infected hamsters demonstrated that their body weights and histopathological findings of the lungs were improved in the yogurt-treated group [49]. Huang and co-workers (2022), via LC-MS/MS, identified 113 peptides released from the major allergens after digestion of yogurt and transportation through Caco-2 cells. Thirty-eight of these peptides existed in all the digested products, among which thirteen peptides had specific biological functions, such as ACE-inhibitory, antimicrobial, and DPP-IV inhibitory properties. Eleven peptides that contained T cell epitopes but not IgE epitopes may induce immune tolerance in CMA; the authors are continuing their work on this topic [50].

Kefir is a dairy product obtained via lactic fermentation using kefir grains, which have bioactive metabolites and bioactive peptides. In previous studies using kefir, the present product has been found to have protective functions in several diseases, such as metabolic syndrome, obesity, hypercholesterolemia, and immune-stimulating effects [51]. The microbiota of kefir is very rich and diverse. Its composition includes lactic fermentation bacteria (e.g., *Lactobacillus brevis*, *Lactobacillus acidophilus*, *Lactobacillus casei*, *Lactobacillus helveticus*, *Lactobacillus lactis*, *Lactobacillus bulgaricus*, *Lactobacillus cellobiosus*, and *Lactobacillus plantarum*), lactococci (e.g., *Lactococcus lactis*, *Streptococcus thermophilus*, *Leuconostoic mesenteroides*, and *Lactococcus cremoris*), and yeasts (*Kluyveromyces*, *Candida*, *Torulopsis,* and *Saccharomyces*) [52]. The use of transglutaminase as a biological glue, during kefir production, allows for perceiving interesting results. The addition of m-TG to fresh kefir resulted in the formation of protein aggregates. Furthermore, two proteins, characterized in 50–55 mL and 135–155 mL of chromatography analysis, were observed, which affected the formation of aromatic acids. During storage, the second fraction (135–155 mL) tended to split into two peaks, both the kefir with m-TG and that without m-TG. Another analysis that involved two-dimensional electrophoresis showed the formation of protein aggregates as an effect of transglutaminase. The final ELISA results from the reaction of kefir proteins with a specific antibody against individual milk proteins (β-lactoglobulin, α-lactalbumin and α-casein, β-casein, and κ-casein) highlighted the difference in protein levels as a result of the addition of m-TG, as well as storage. The immunoreactive β-lg was not detectable in the kefir samples and was under the accepted ELISA conditions [53]. The animal experiment carried out with Brown Norway rats of BN/SsNolaHsd sensitized to dairy products allowed us to observe that the developed kefir product, produced with the use of whey protein hydrolysate, was characterized by hypotensive, hypolipidemic, and hypocholesterolemic effects compared to kefir produced using traditional technology and reduced the allergenicity of β-lactoglobulin, the main milk allergen [54].

The richness of the kefir microbiota has long been of interest to scientists. Now, due to metagenomic sequencing analysis, it has been possible to reveal the composition of the bacteria. The microbiota of Tibetan kefir strains showed the presence of 522 species, with *Lactobacillus kefiranofaciens* as the most abundant species. *Lactobacillus* and *Bifidobacterium* were the most diverse probiotics. Analysis after 8 h, 24 h, and 36 h of fermentation identified the different metabolites revealed at successive times of the process. Knowledge of the seven metabolic pathways mainly related to lipid metabolism, biosynthesis of amino acids, carbohydrates, and other chemical compounds, and biosynthesis of phenylpropanoids were enriched, as well as the eleven pathways related to amino acid metabolism, the citrate cycle (TCA cycle), translocation, membrane transport, and biosynthesis of secondary metabolites [55].

According to recent European research trends and the needs of consumers, by-products of the dairy industry, such as whey and buttermilk, are considered valuable raw materials for the production of high-value products [56,57,58]. Both of them are sources of nutrients and bioactive compounds, and are suitable (from an economical point of view) to produce new, immunomodulating beverages.

**Figure 1:**
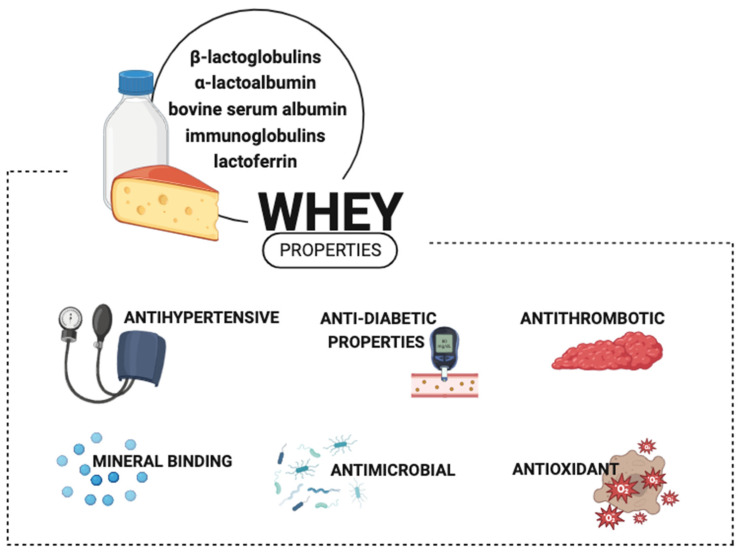
Pro-health benefits of whey. Created using BioRender.com (accessed on 5 December 2023).

Whey is a by-product of cheese production in the dairy industry (Figure 1). It represents a high concentration of organic compounds. Whey contains water (93%), carbohydrates (mainly lactose, about 5%), proteins (0.85%), fats (0.36%), minerals, and soluble vitamins [59,60,61]. Proteins present in cow milk whey belong to strong allergens, e.g., β-lactoglobulin (β-lg) and α-lactalbumin (α-la), which account for 55–60% and 15–20% of total proteins, respectively. β-Lg is one of the main cow’s milk proteins, but it is absent in human milk and rodents. Whey includes bioactive immunoglobulins, lactoferrin, lactoperoxidase, growth factors, and bovine serum albumin. The denaturation and proteolysis of proteins allow for an increase in their digestibility; therefore, whey protein hydrolysates are physiologically more beneficial for the organism than intact proteins, as their intestinal absorption becomes more efficient [62].

Studies that have been carried out with the BALB/c mouse model showed a distinct difference in the influence of whey protein hydrolysates and whey in the prevention of intestinal inflammation and the development of food allergies. The authors used Peptigen IF-3080, a hydrolysate with a degree of hydrolysis of 29% (Arla Food Ingredients, Viby J, Denmark), and 80% whey protein concentrate. The obtained results indicated that the whey hydrolysate prevented intestinal inflammation and the clinical manifestation of food allergies in sensitized mice. The ability of BLG to induce allergic inflammation was decreased. Oral administration with hydrolysates maintained levels of sIgA, IL-4, and IL-5 similar to the control group. The authors suggested that whey protein hydrolysates can be safely used by already sensitized children [63].

The technological production details of commercial hydrolysates, as well as their chemical composition, are usually the property of the company and are not disclosed to the general public.

A research model for whey fermentation using yogurt starters and the addition of potentially probiotic bacteria has been proposed. Modulation of the host immune response with fermented whey was carried out in a mouse Balb/c model. The fermentation procedure was carried out with a few bacterial strains (*Streptococcus salivarius* ssp. *thermophilus* 2K, *Lactobacillus delbrueckii* ssp. *bulgaricus* B, *Lactobacillus bulgaricus* BK, *Lactobacillus plantarum* W42, and *Bifidobacterium animalis* ssp. *lactis* Bi 30), and the immunomodulatory potential of beverages was determined. The presence of *Lactobacillus plantarum* W42 and *Bifidobacterium lactis* Bi30 next to the yogurt starter bacteria significantly enhanced the health effects of whey beverages. The results showed that the Th1/Th2 balance was altered towards the Th1 path response. The secretion of regulatory cytokines (IL-10 and TGF-β) was significantly increased. The fermentation procedure caused a reduction in allergy marker levels (IL-4, IgE, and IgG1-specific levels) [64].

Buttermilk is a by-product of butter production and is also applied to bakery products or various dairy products [65]. Its composition comprises proteins (31.5–33.1%), lactose (48.7–53.8%), and fats (5.7–13.1%). Buttermilk is mainly processed into powder, which has limited functional properties. It consists of an aqueous phase, the cream, separated from the butter mass produced when it is whipped. Its rich chemical composition and unique structure can provide many functional ingredients, such as stabilizers, emulsifiers, and health organizers, for many food products [66]. The unique composition of buttermilk contains a membrane of milk fat globules and phospholipids, which have been shown to have beneficial effects on the proper functioning of brain health and cognitive development in early infancy [67]. The MFGM (milk fat globule membrane) is a 3-layer membrane structure that is composed of proteins, non-polar lipids, (62% triglycerides and 9% diglycerides), and polar lipids (26–31% phospholipids). The three layers in the MFGM are monolayers, which are located in the interior and mainly have polar lipids, such as phosphatidylethanolamine (PE), phosphatidylinositol, and phosphatidylserine. The protein layer and outer bilayer, on the other hand, are composed of phospholipids, proteins, polar and non-polar lipids, and enzymes [68]. The impact of buttermilk, mainly choline and its metabolites, on the animal immune system feeding the Sprague–Dawley rat pups during lactation and weaning periods was determined [69]. After 3 weeks of the experiment, the results showed an increased level of TNF-α and IFN-γ. LPS stimulation enhanced the production of IL-10 and TNF-α. Starting at week 6 of age, a higher body weight of rats was observed. ConA stimulation caused the release IL-2, IFN-γ, and IL-6. Choline induces modulated T cell function and forms higher Th1 than Th2 responses. The production of the cytokine IL-10 after LPS stimulation was more intensive, which suggested an anti-inflammatory response. The study proved that the lipid-soluble forms of choline are important for the development of the immune system, and that buttermilk can develop the immune system early in life. The studies carried out with the volunteers showed that after consumption, the enrichment of a fermented buttermilk beverage with an addition of 0.3% milk protein concentrates (1.2% of fats, 86.5% of proteins, and 3.5% of lactose, JSC MG Baltija, Medeikiai, Lithuania) over a 21-day period did not show any statistically significant effect on the biochemical blood parameters of the volunteers. A reduction in the mean values of lipid levels in plasma was noticed [70]. Due to the content of allergenic proteins in buttermilk’s chemical composition, a study was undertaken that looked at the use of the *Lactobacillus casei* LcY strain, which reduced the immunoreactivity of milk proteins in the fermented raw material. The results proved that the fermentation of sweet buttermilk with the *Lactobacillus casei* LcY strain essentially reduced the immunoreactivity and allergenicity of the main whey proteins present in cow’s milk. A three-step simulation digestion process of fermented buttermilk was undertaken, resulting in an additional reduction in the immunoreactivity of the allergens tested (α-lactalbumin, β-lactoglobulin, α-casein, β-casein, κ-casein, bovine serum albumin, and lactoferrin). Cyclopropane fatty acid synthase, phospholipid synthase, and carboxylate-amine ligase present in the *Lactobacillus casei* LcY proteins have been documented to react with human IgE antibodies. This shows that some of the proteins present in the lactobacilli may have allergenic properties, which should be taken into account when designing new fermented products [71].

Whey and buttermilk beverages, fermented and non-fermented, with the addition of fruits, vegetables, and juices made thereof, spices, and honey, are of interest to many consumers. However, an important issue to be worked out is how protein components combine with, for example, polyphenols present in food additives and how they are digested in the body at a molecular level and point to the health advantages of immunomodulating products. Pointing to the health advantages of immunomodulatory products, the dairy market will enhance its proposal of new products. In addition to cow’s milk, the milk of other animals may be consumed, although this is a rather marginal production with a limited geographical scope.

Mare’s milk has been consumed since the dawn of time, mainly in Central Asia and in neighboring regions of this continent. The composition of this type of milk is very similar to that of breast milk but differs significantly from bovine milk [72]. The high lactose content allows for extremely good growth of the intestinal microbiota. Mare’s milk is characterized by a high content of lysozymes and lactoferrin and contributes to the inhibition of pathogenic bacteria [73]. Mare’s milk characterizes more whey proteins than caseins [74]. An experiment carried out in mice by the team of Fotschi et al., 2016 [75] showed the immunomodulatory properties of mare’s milk. The obtained results showed a positive effect of consuming mare’s milk on reducing IgE levels in immunized animals and on the associated increase in the number of Treg cells, which play an immunosuppressive role. As a result, mare’s milk results in a positive effect on the innate response, which promotes a decrease in IL-4 expression and an increase in TL-4 expression, with no effect on the condition of the animal [75]. The beneficial changes in the composition of the intestinal microbiota were inferred to be exerted by an increase in *Bifidobacterium* spp. in the intestinal environment. Comparing the biochemistry parameters, immunity indicators, T cells, gut microbiota abundance, and metabolites of cow, goat, and mare milk were the most similar to human milk and can positively modulate the gut microbiota and immunity status of infants. The authors suggested that mare’s milk could replace human milk [76].

The fermented milk product is kumis, a lactic–alcoholic beverage made with *Lactobacillus delbrueckii* ssp. *bulgaricus*, *Lactobacillus casei*, *Lactococcus lactis* ssp. *lactis*, *Kluyveromyces fragilis*, and *Saccharomyces unisporus*. In the chemical composition determined the presence of lactic acid (7–18 g/kg), ethanol (6–25 g/kg), and carbon dioxide (5–9 g/kg) due to spontaneous fermentation [77]. Kumis is extremely tolerable by people with an intolerance to cow’s milk, atopic dermatitis, and chronic diseases of the gastrointestinal tract.

It is important to note that fermented milk beverages (e.g., yogurt, kefir, whey, buttermilk, and kumis) produced with appropriately selected strains are characterized by reduced immunoreactivity compared to their starting raw materials.

## 3. Bioactive Milk Products with Pro-Health Properties

Studies on the effects of fermented beverages on the host body have indicated their immunomodulatory potential. Hence, there has been a strong development of research towards defining immunoreactive molecules and their targeted interactions. In recent years, numerous studies have been carried out proving the biological effects of active milk peptides originating from different animals on body protection and treatment in various diseases [78,79,80,81,82,83,84].

Bioactive peptides are categorized as characteristic protein fragments with a range of biochemical and health-related properties. In terms of their behavior, they may resemble medicines and thus have a protective function, reducing the adverse effects of non-communicable chronic diseases possessing antihypertensive, immunomodulatory, antioxidant, and opioid properties. The functions of peptides are associated with their amino acid composition and content [85]. They typically consist of between 2 and 20 amino acid residues and are encoded in the primary structure of plant proteins and animal proteins as an inactive form [86]. In a study carried out by Ong and Shah (2008), it was shown that Cheddar cheeses that were made with the use of the probiotics *Lactobacillus casei* 279, *Lactobacillus casei* LAFTI^®^ (Ostfildern, Germany) L26, or *Lactobacillus acidophilus* LAFTI^®^ L10 were shown to exhibit significantly higher levels of ACE-inhibitory activity compared to those without any probiotic additives. ACE-inhibitory peptides were identified as κ-CN (f96-102), αs1-CN (f1-9), αs1-CN (f1-7), αs1-CN (f1-6), αs1-CN (f24-32), and β-CN (f193-209). They were mostly released during the early stage of ripening, and some of them were hydrolyzed into smaller peptides during proteolysis [87]. Cavalheiro (2020) and her research team proposed a yogurt product with the addition of *Lactobacillus helveticus* LH-B02. Using tandem mass spectrometry, they confirmed the presence of two bioactive peptides, namely αS1-CN (f24-32) and β-CN (f193-209), which arose due to the addition of LH-B02 [88].

The milk of other animals (e.g., goat, sheep, camel, and donkey) has also been analyzed in terms of their pro-health properties. In a study conducted by Ashokbhai et al., 2022 [89], the antioxidant, anti-inflammatory, and antimicrobial properties of fermented sheep milk using *Lactobacillus fermentum* KGL4 cultures were evaluated. The antioxidant and antimicrobial peptides were determined (ITMPLW, FAWPQYLK, HKEMPFPK, LDQWLCEK, and KADEKKFW) and confirmed using the BIOPEP database. The antimicrobial activity of fermented sheep milk against *Enterococcus faecalis*, *Salmonella typhimurium*, *Bacillus cereus*, and *Escherichia coli* was also observed. The study with LPS-activated RAW 264.7 cells showed that fermented sheep milk with KGL4 significantly reduced the excessive levels of pro-inflammatory cytokines, e.g., TNF-α, IL-6, and IL-1β [89].

Fermented camel milk is a valuable source of bioactive peptides with antioxidant activity. Dharmisthaben (2021) and colleagues used the strain *Lactobacillus plantarum* KGL3A for camel milk fermentation, which allowed them to obtain new antioxidative peptides; however, the authors emphasized that clinical trials should be carried out to confirm the other biological activities [90].

Donkey milk has been established as a nutraceutical, and it was shown that the LAB fermentation process could enhance its value. The bacterial strains *Lactobacillus rhamnosus* 17D10 and *Lactococcus lactis* ssp. *cremoris* 40FEL3 were selected to carry out the fermentation of donkey milk. The antioxidant, antibacterial, and antiviral activities of the peptide mixture were analyzed, and it was characterized with LC-MS/MS. It has been proven that only the peptides produced by *Lactobacillus rhamnosus* 17D10 were able to reduce *Staphylococcus aureus* growth. All the peptide mixtures were able to inhibit the replication of HSV-1 by more than 50%. Seventeen peptides were found to have 60% sequence similarity with already known bioactive peptides. These authors showed that the lactic acid fermentation of donkey milk enhances the value of milk [91].

Currently available milk-derived products containing bioactive peptides comprise whey protein isolates, low-fat cheese, chewing gum, soft milk drinks, fermented milk, and milk ingredients used for the supplementation of food. Many peptides as milk protein fragments were determined, and they have been deposited in the internet databases [92], and the researchers still isolate new bacterial strains from lactic acid bacteria species with beneficial pro-health properties. The literature data have drawn attention to the conditions of fermentation (e.g., time and temperature presence of diverse substrates) and the detailed, comprehensive characterization of the obtained peptide pool. Italian researchers have analyzed several LAB strains; among them, *Lactobacillus helveticus* exhibited the most beneficial proteolytic activity. During further analysis, its fermentation activity was monitored via reversed-phase high-performance liquid chromatography and nano-high-pressure liquid chromatography coupled with a high-resolution mass spectrometry analyzer (nano-HPLC-HRMS) with the aim of the preliminary identification of fractions from fermented skim milk. Such an analytical procedure has made it possible to observe and influence the optimization of the fermentation parameters during the retrieval of bioactive peptides [93].

**Figure 2:**
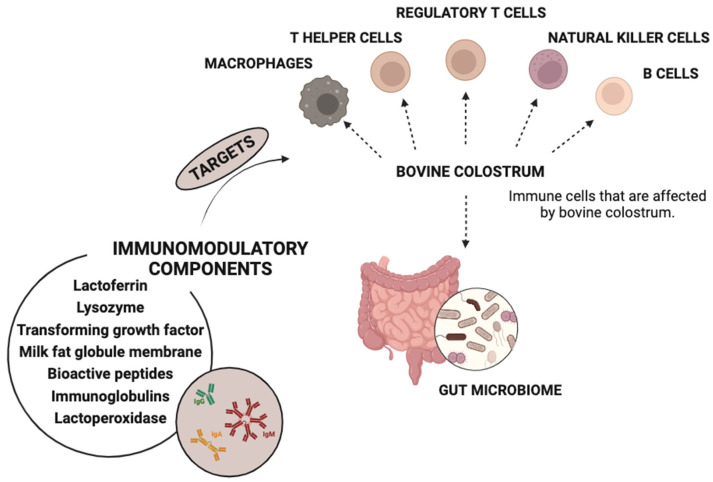
Immunomodulatory properties of bovine colostrum. Created using BioRender.com (accessed on 5 December 2023).

Colostrum is a well-known source of bioactive peptides with immunomodulatory properties (Figure 2). A study focused on the comparative antimicrobial properties of buffalo bioactive peptides generated with the use of *Lactobacillus rhamnosus* C25-, *Lactobacillus rhamnosus* C6-, and *Lactobacillus casei* NCDC17-fermented colostrum whey. Peptide fractions of 3 kDa, 5 kDa, and 10 kDa and their antimicrobial activity against diarrheagenic *Escherichia coli* strains were evaluated. Higher levels of inhibition were demonstrated by the peptide fractions of less than 10 kDa from *Lactobacillus rhamnosus* C25-fermented colostrum whey, and the authors stated that such a product can be used as an ingredient in functional foods against diarrhea [16].

Many factors (e.g., aging populations, chronic diseases, such as metabolic diseases, cardiovascular diseases, mental health problems, and cancer disorders, stress, and a modern style of life) consist of the fact that work on new, fermented products continues and new knowledge of their bioactive activity will be increasingly desirable to realize informed consumption. Food manufacturers are looking for modern products with attractive properties that are not only functional but also support immunity and activate antiaging and anti-inflammatory processes (Table 1). It is important to obtain new knowledge about the immunomodulatory properties of milk-polyphenolic and anthocyanin products as carriers of adducts with cumulative bioactive characteristics. An indication of the potential synergy of ingredients is required for the evaluation of new food products.

It is important to note that the milk of another animal than cows (e.g., goats, sheep, camels, donkeys) and colostrum possess pro-health, immunomodulatory properties.

## 4. Polyphenols as Valuable Food Additives

The introduction of new, attractive products to the market represents the development of the food industry, develops competitiveness, and encourages consumers to buy products with proven quality and functionality. Customer expectations are directed towards food additives with a health-promoting character. One of the most popular groups of substances is bioactive polyphenols (e.g., flavonoids, phenolic acids, lignans, and stilbenes), which have been perceived as reducing the risk of chronic diseases that include cancer, diabetes, cardiovascular and neurodegenerative diseases, infectious illnesses, or inflammatory disorders [98,99]. Polyphenols are natural compounds produced by plants [100]. Their presence provides plants with protection from UV radiation [101], microbes or herbivores [102], and also attracts pollinating insects [103]. People come into contact with polyphenols in foods, e.g., fruits and vegetables, as well as in drinks, such as red wine, tea, or coffee [104,105]. Thus, polyphenols affect their taste, smell, or color as they have in their structure a minimum of one aromatic ring containing more than one hydroxyl group [106,107]. A lot of the data that have been presented in the literature have described the properties of polyphenols, their natural sources, the daily intake dose, their association with outcomes, and their overall results, depending on gender, age, physiological state, and other factors, as effects of selected plant bioactives on inflammatory markers and intestinal immunity [108,109,110,111]. The origin of the source of phenols and the amount of their consumption depends on the geographical area and dietary habits.

Determining the safe daily intake of polyphenols and/or their metabolites is still a topic of research. To date, analysis of available results by EFSA experts has identified sufficiently studied olive polyphenols [112] and green tea catechins [113]. According to the opinions of the EFSA, the use of health claims related to polyphenols in the EU market and in the “case law” of the EFSA’s opinion is only limited to “olive oil polyphenols” with the following statement: “Olive oil polyphenols contribute to the protection of blood lipids from oxidative stress.” The beneficial effect is obtained with a daily intake of 20 g of olive oil. All other formulations related to other foods have been rejected. The EFSA rejected health claims regarding “berries (lingonberry, cloudberry, blueberry, currants, raspberry, and strawberry)” and their presumed “protection of DNA, proteins, and lipids from oxidative damage”. The general opinion is that “Natural berries contain plenty of natural antioxidants (polyphenolic compounds, vitamin C, and carotenoids) and fiber but only a small amount of energy and sodium. For this reason, they are very suitable for a heart-friendly diet” [112]. Therefore, various studies are being undertaken to indicate the direction of the effects of individual polyphenols on the body, under physiological and pathological states, and to determine their optimal doses. Numerous studies regarding catechins provided scientific data prepared regarding their properties, dosages, and activity. The EFSA Scientific Committee gathered the data concerning the adverse effects of green tea catechins on the liver and reported that the intake of doses equal to or above 800 mg of EGCG per day induces a statistically significant increase in serum transaminases in treated subjects compared to their controls. However, in the clinical studies that have been reviewed, there is no evidence of hepatotoxicity below 800 mg of EGCG/day up to 12 months. The experts have recognized that it is plausible that the kinetics, as well as the toxicity of green tea catechins, could be modified through the matrix in which they are present [113]. Polyphenols are present in plants (vegetables and fruits) and can be added to foods as supplements isolated and dried or lyophilized. Del Bo et al., 2019 [111] suggested the daily dose of polyphenols should be about 900 mg, and pointed out that an intake above 1170 m/day exhibits beneficial effects on cardiovascular risk and mortality [111].

The setting of the optimal dietary daily dose of polyphenols is problematic due to the variable polyphenol content in raw materials and products thereof, even within a specific food item. Factors determining the number of polyphenols in fruits and vegetables include the annual climate, post-harvest processing, technological processing, and storage conditions [114]. The bioavailability of polyphenols is changing in line with the gut digestion transit of food during hydrolysis, interactions with bacterial metabolites, and chemical reactions, like glycosylation, conjugation, and polymerization. Most of the literature data describe potential health benefits and properties as antioxidant, anti-inflammatory, anticancer, cardio-protective, anti-neurodegenerative, and antidiabetic [115,116,117]. However, there is also the opposite information: some adverse effects are possible due to the high doses of polyphenols through prooxidative effects. The well-known samples from grapefruit inhibit the enzyme thyroid peroxidase, reduce fertility, retard sexual maturation, induce iron complexation, and inhibit the drug-metabolizing enzymes naringenin, naringin, and 6,7-dihdroxybergamottin [114,118,119].

As rich sources of polyphenolic compounds, both phenolic acids and flavonoids are in berries, such as chokeberries, strawberries, raspberries, blueberries, acai, goji berries, cranberries, blackberries, and others, occurring locally, e.g., Indian gooseberry (*Phyllanthus emblica*), Ceylon gooseberry (*Dovyalis hebecarpa*), and Deerberry (*Vaccinium stamineum*) [108]. Among these berries, black chokeberries (*Aronia melanocarpa*) deserve special attention, with the highest content of anthocyanins and other polyphenols among plant materials. The chokeberry fruit contains both components with antioxidant properties, including anthocyanins, flavonols, phenolic acids, and tannins, as well as vitamins (C, B2, B6, E, P, and PP) and minerals (Mo, Mn, Cu, B, I, and Co). The most important components of chokeberry fruit, responsible for most of its beneficial health properties, are polyphenols, the contents of which (depending on the variety, growing conditions, and harvest season) vary from 2000 to 8000 mg/100 g dry weight [120]. It has been stated that the fruit of chokeberry does not accumulate heavy metals, such as cadmium, lead, arsenic, and tin. Chokeberry fruit has been commonly used for making juices and syrups, teas, dries, jellies, jams, tinctures, and wine and enriching foods in valuable bioactive ingredients. Chokeberry fruit pomace, remaining after the juice pressing process, can be a valuable raw material for obtaining anthocyanin pigments; however, the substance that potentially limits the use of pomace as a food or animal food additive is amygdalin [121].

The research-confirmed potential of polyphenols to affect the human body makes them a valuable dietary supplement. However, in several cases, there is also a risk associated with their overdose, leading to the need to implement research in this area. An increase in the total contents of flavonoids and polyphenols during the spontaneous fermentation process was demonstrated. The tested juices of cruciferous (*Brassica*) vegetables showed relatively low contents of antioxidants per gallic acid. In contrast, the fermentation leachate fermentation contained measurably higher amounts of phenolic compounds. Vegetables with a high amount of polyphenols in their composition are also known and valued. The 80% aqueous methanol extract of sauerkraut was characterized by a higher content of total phenolics (8.25 mg/g) than that of white cabbage (5.72 mg/g). Phenolic compounds present in extracts showed antioxidant and antiradical properties. The total antioxidant capacity of the sauerkraut extract (0.031 mmol Trolox/g) was stronger than that of white cabbage (0.025 mmol Trolox/g) [122].

Beetroot and its products are the main sources of phenolic acids and flavonoids, with predominating isoferulic acid, protocatechuic acid, epicatechin, and apigenin. Polish research showed that the spontaneous fermentation process caused an increase in the content of free phenolic acids and reduced the content of conjugated phenolic acids. Contrarily to phenolic acids, the same process caused a reduction in the content of free flavonoids and an increase in the content of conjugated flavonoids. Moreover, the 14-day spontaneous fermentation process resulted in a significant reduction (by 45.18%) in the total content of phenolics (phenolic acids and flavonoids). Fermented beetroot juice in a dose of 200 mL/60 kg body weight was consumed by forty-two days. It was observed that the long-term and regular consumption of red beetroot affects the profile and concentration of phenolics in the blood plasma and urine of volunteers [123].

New information about unconventional raw materials for polyphenols is now appearing in scientific reports all over the world. The properties of a popular Brazilian berry, the jaboticaba fruit, was described [124]. It was found and defined as having than 80 polyphenol compounds, including anthocyanins, hydroxybenzoic acid derivatives (e.g., ellagotannins, gallotannins, and ellagic acid derivatives), hydroxycinnamic acids, flavonols, flavanols, flavanones, and flavones. The highest contents were found in the seeds and the skin of the jaboticaba fruit. The peel is an excellent source of anthocyanins (mainly cyanidin-3-*O*-glucoside) and hydrolyzable tannins (mainly vescalagin, castalagin, and pedunculagin), while the seeds are rich in hydrolyzable tannins. In an experiment with rats, the powder and aqueous extract of jaboticaba peel containing cyanidin-3-*O*-glycoside and ellagic acid were used. The jaboticaba extract treatments reduced weight gain and adiposity, improved insulin sensitivity, increased HDL cholesterol, and prevented hepatic steatosis. These results depicted that such a supplementation can modulate the parameters of obesity and insulin metabolism, preventing liver steatosis in obese hosts [125].

Plant raw materials containing polyphenols are a valuable addition to food products. An example is bread made with sorghum flour. Red sorghum is a gluten-free cereal with a high content of nutrients, polyphenols, and bioactive compounds with potential functional and health benefits. The use of sorghum flour contributed to a significant (*p* < 0.05) enrichment of the bread in protein, dietary fiber, and phenolic compounds that increase its antioxidant power (ABTS, DPPH, FRAP, and CUPRAC). The addition of 30% and 40% sorghum flour improved the technological parameters, such as specific volume, crumb quality, and color, which was confirmed via consumer sensory analysis and was designed as a new product with a high bioactive polyphenol level [126].

Prebiotics are a group of compounds that contribute to the maintenance or restoration of the normal gut microbiome [127]. Among the new generation of prebiotics are polyphenols, which, as potential “metabolic prebiotics,” can contribute to stimulating the growth of beneficial bacteria and inhibit pathogenic bacteria (Table 2) [128]. The prebiotic properties of polyphenols will vary depending on their source and chemical structure, as well as the individual composition of the intestinal microbiota [128,129]. Studies on the correlation between the polyphenols and the composition of the intestinal microbiota have largely focused on flavonoids and their subclasses [130]. In vitro studies conducted by Zhu et al., 2018 [131] proved that anthocyanins and anthocyanin monomers from black rice exhibit prebiotic effects by inducing the proliferation of *Bifidobacterium* and *Lactobacillus* species [131]. Similar results were presented by Zhang et al., 2016 [132], with purple sweet potatoes being the source of the anthocyanins. These researchers showed that the presence of these anthocyanins additionally inhibits the growth of *Bacteroides-Prevotella* and *Clostridium histolyticum* [132]. In vivo studies conducted on mice also confirmed the positive effects of anthocyanins. Supplementation with anthocyanins derived from dark sweet cherry powder caused changes in the intestinal microbiota of the animals’ colon, as manifested by higher levels of *Akkermansia* spp. [133]. An increase in the same group of bacteria was observed with the use of cranberry extract containing anthocyanins and proanthocyanidins, which further reduced triglycerides in the intestines of animals and decreased oxidative stress and intestinal inflammation [134]. A human-based intervention study showed similar results. Daily consumption of a beverage with a high content of cocoa flavonols for 4 weeks increased the amount of bacteria from the *Lactobacillus* spp. and *Bifidobacterium* spp. groups compared to the control, where a beverage with a low content of cocoa flavonols was employed. The prepared beverages had no other substances in their composition that could be attributed to prebiotic properties; hence, the effect was attributed to the cocoa flavonols. Confirmation of their prebiotic properties was provided by in vitro studies, where incubation of fecal microbiota with flavanol-enriched cocoa extract increased the amounts of these bacteria [135]. Polyphenols derived from blueberry extract (PPE) applied to obese C57BL/6J mice inhibited weight gain and restored normal lipid metabolism. Using sequencing of the 16S rRNA gene of fecal microbiota, it was shown that supplementation with this extract caused modulation of the composition of the intestinal microbiota [136]. As previous studies have demonstrated, wine is also a good source of polyphenols with prebiotic effects. In an in vitro study, residues of red and white grapes caused an increase in *Lactobacillus* and *Bifidobacterium* [137]. In vivo studies conducted on rats also indicated an increase in beneficial *Lactobacillus* bacteria in response to the presence of a non-alcoholic extract of red wine polyphenols, which included anthocyanins, monomeric flavanols, flavonols, and phenolic acids, as well as complex phenols and tannins with various degrees of polymerization. A decrease in *Propionibacterium*, *Bacteroides*, and *Clostridium* and an increase in *Bifidobacterium* in the contents of the colon were also observed, indicating protection against the carcinogens that were used in the study [138].

It is important to note that polyphenols (e.g., phenolic acids and flavonoids) present in fruit and vegetables are considered to be antioxidant, anti-inflammatory, anticancer, cardio-protective, anti-neurodegenerative, antidiabetic, and antiradical compounds of food and are perceived as prebiotics.

## 5. Protein–Polypeptide Interactions

Both polyphenols and milk proteins are seen as bioactive food ingredients. They are used as functional additives with immunostimulatory properties. Therefore, it seemed reasonable to combine them in order to multiply the beneficial properties of the food matrix. An overriding aspect of the development of protein–polyphenol mixtures was their technological properties and their suitability for consumption over a longer period of time than the individual components [139,140]. Buyers demand an attractive new commodity, while the food industry demands a reduction in waste and the provision of economic benefits. Phenolic compounds interact with proteins through two different mechanisms: non-covalent interactions and covalent interactions. Non-covalent interactions are reversible and comprise hydrogen bonding, hydrophobic interactions, electrostatic interactions, and van der Waals forces. Covalent interactions between proteins and phenolics are characterized by the formation of covalent bonds under enzymatic and non-enzymatic procedures [141]. The detailed mechanism was described in a number of literature data [142]. Combining milk proteins with polyphenols depends on their structure, mutual interactions, and reaction conditions.

The combination of milk proteins with polyphenolic compounds was first observed over 60 years ago. The breakthrough was to determine the ratio of proteins to polyphenols (tannins) to avoid precipitation of the protein components in the mixture. It was found that at a pH close to the isoelectric point of the protein, the weight ratio of proteins to tannins resulting in the complete precipitation of the protein was 1:0.5. At lower tannin concentrations, the protein was only partially precipitated, and at tannin concentrations 10 times lower than the protein, precipitation did not occur [143].

The interactions between polyphenols and milk proteins have been described. The individual components can combine as a result of covalent and non-covalent interactions. Covalent, irreversible bonds are formed due to enzymatic, thermal, and alkaline conditions, and the oxidative formation of *o*-quinones also reacts with the nucleophilic groups of proteins [144,145,146]. More common, non-covalent, reversible binding between proteins and polyphenols include the van der Waals forces, electrostatic interactions, and hydrophobic reactions, usually stabilized with H-bonds. Hydrophobic interactions have been observed between polyphenols’ aromatic benzenoid rings and proteins’ hydrophobic regions. Hydrogen bonds have been found between the hydroxyl groups of the polyphenols and the polar amino acids of the proteins [147].

One of the most important issues in the area of food technology is the assessment of food safety, with particular emphasis placed on the allergenicity of new products. The production of protein–polyphenol mixtures fits into the global trend; therefore, the conditions for optimizing technological parameters are being developed. So far, several examples of studies on the mutual interactions of allergens and polypeptides have been presented.

Pessato and co-workers (2018) determined the structural changes of whey proteins combined with caffeic acid and (−)-epigallocatechin gallate. The analysis of the obtained complex proved lowering the allergenicity by reduced IgE-binding capacity to the strong milk allergens β-lg and BSA [29]. Similar results were obtained by Wu, with their research group (2018) having studied the functional properties of bovine β-lactoglobulin after covalent conjugation with (−)-epigallo-catechin 3-gallate and chlorogenic acid. The covalent bond between the polyphenols and the amino acid side chains of βLG was confirmed via MALDI-TOF-MS and SDS-PAGE. Structural analysis via fluorescence spectroscopy, circular dichroism, and Fourier-transform infrared spectroscopy confirmed covalent conjugation. The ELISA method showed reduced IgE binding with β-lactoglobulin [148]. Both studies presented a promising approach to producing novel foods with reduced allergenicity for vulnerable groups of consumers.

The covalent and non-covalent interactions between cyanidin-3-*O*-glucoside and two milk-allergenic proteins, α-casein and β-lactoglobulin, were estimated during simulated digestion. The hydrolysis of mixtures during simulated digestion proved that the covalent complexes exhibited reduced protein digestibility, but not their non-covalent counterparts. The same mixtures were observed to lower IgE-binding levels in comparison to the native control. The anthocyanin–milk protein product can be proposed as a functional ingredient with health-beneficial properties [149].

The covalent interaction between polyphenols (epigallocatechin gallate, procyanidin, apple polyphenol, and puerarin) and whey protein isolates showed rheological changes as well as improved foaming and emulsifying properties of the whey proteins; however, its surface hydrophobicity was greatly decreased. The particle sizes of conjugates and their surface were changed, which were observed via scanning electron microscopy [30].

Interactions between polyphenols and proteins cause many effects, like stabilization of proteins, decrease in the antioxidant activities of proteins and polyphenols, reduction in protein digestibility, increase in thermal stability, modifying the physiochemical properties, and many others, as described in the data that has been presented in the literature [150,151,152].

Han’s study found that phenolic compounds interacted with casein rather than with whey proteins. Size-exclusion chromatography analysis found that the aggregates of casein and the phenolic compounds from an extract of green tea, grape, or cranberry were bigger than with whey proteins. Not only pure phenolic compounds (EGCG and tannic acid) but also natural crude polyphenols (green tea, grape, and cranberry extracts) demonstrated strong hydrophobic binding with calcium caseinate. Whey protein isolate (WPI) and polyphenols hardly interacted [153]. The interactions, on a molecular basis, between food components and analyzed in vitro with cell lines and in vivo models are crucial for finding the most favorable solution for consumers, especially for the so-called vulnerable groups (e.g., people with digestion tract diseases, food allergies, pregnant women, and elderly consumers). Dairy products derived from the milk of various animals (cow, goat, sheep, buffalo, etc.) are usually the first food for newborns and an integral part of the human diet. In contrast, the predominant sources of polyphenols are varied in different parts of the globe, and their occurrence is geographically dependent and popular among local consumers.

Black carrot is a common Indian cheap root vegetable easily cultivated during the whole year, rich in polyphenols, and its mineral content possess antioxidant potential. To meet the challenges facing India (lack of wholesome food, malnutrition, hunger, etc.), it has been proposed to use black carrots as an additive to dairy products, which have numerous health benefits, to encourage consumer acceptance of a new product. The Punjab Black Beauty variety of Indian black carrots (*Daucus carota* L.) was proposed as a 7.5% concentrate in the development of acceptable dairy products, like ice cream, yogurt, and buttermilk. The analyses revealed increased total anthocyanin, phenols, flavonoids, antioxidant activity, magnesium and iron content of products, and good sensory properties [154]. The storage conditions allowed one to observe that ice cream could be stored for more than 60 days, yogurt for up to five days, and buttermilk for up to 10 days with extremely good stability properties.

The application of 0.25–0.75% cranberry pomace to milk prior to fermentation resulted in a significant increase in protein particle size, in addition to accelerating the gelation time of milk depending on the concentration of cranberry pomace addition due to the complexation of polyphenols with milk proteins. The protein–polyphenol interaction was responsible for the formation of aggregated protein networks with larger particles, higher viscosity, and lower syneresis in yogurt made with cranberry pomace compared to yogurt made without cranberry pomace. However, the use of the extract had no effect on the viability of the yogurt bacteria. Yogurt with cranberry pomace exhibited significantly slower protein digestion than the control, in addition to a limited release of phenols at the end of intestinal digestion [155].

Combining milk with fruits and lactic acid fermentation requires attention to many variables when setting the conditions of the planned process. Conditions such as the time of the reaction, temperature, weight ratio (milk proteins: polyphenols ratio), concentration of proteins and polyphenols, structural composition, and pH of the mixture are crucial in obtaining attractive products. Tannins present in fruits can precipitate the proteins from the solution [156,157]. The extremely important parameter is the selection of bacterial strains. *Lactobacillus casei* CECT 475, *Lactobacillus paracasei* subs. *paracasei* CECT 277, and *Lactobacillus helveticus* CECT 541 were selected as starters for the fermentation of cow’s milk with sweet cherry puree. The temperature of the processes was varied (30 °C for *Lactobacillus paracasei*; 37 °C for *Lactobacillus casei* and *Lactobacillus helveticus*). The samples were stored for up to 10 days. The studies showed that fermented milk with *Lactobacillus helveticus* provided the best preservation of anthocyanins. The stage of maturity of fruits affects their color, nutrition, and health-related properties [158]. Milk that originated from other animals, e.g., sheep, was also employed to produce the fermented product. The milk was fermented with *Lactobacillus acidophilus* LA-5^®^ and *Lactobacillus rhamnosus* Pen E/N Oxy^®^ (Houston, TX, USA) and combined with the chokeberry fiber as a prebiotic. The addition of fiber to sheep milk before fermentation stimulated the growth of bacteria. The new products were characterized as having a more intense additive taste and acidic flavor. The authors also pointed out an undesirable feature of the product; the addition of chokeberry fibers increased syneresis in each fermented product, regardless of the bacteria used for fermentation. The fiber intensified the sour taste and the taste of the additive in both types of fermented sheep milk. It was observed as a significant reduction in brightness, an increase in red color, and a decrease in the yellow color of the fermented products. The fermentation with *Lactobacillus acidophilus* caused the formation of a harder gel than with *Lactobacillus rhamnosus* [159].

It is important to note that proteins and polyphenols can bind in complexes via non-covalent or covalent interactions, depending on the conditions of the reaction environment.

## 6. Protein–Polyphenol Products

Proposing new milk protein–polyphenol products involves the issue of food safety, where product allergenicity plays an important role. Lila (2017), with her research group, described the studies with the use of WPI and cranberry, muscadine, and blackcurrant juice concentrates. The immunoreactive properties of the interacted milk proteins–polyphenols were determined with the levels of cytokines revealed from the macrophages and dendritic cells. The authors observed the reduction in immune responses to allergenic proteins. They estimated that the suppressive effect can be the result of binding polyphenols with proteins, preventing the proteins from binding to their appropriate receptors on macrophages or dendritic cells. A second solution to this issue has also been put forward, indicating that the polyphenols can react with cell surface receptors, e.g., receptors not binding to the allergenic protein. The experiment with porcine macrophages and dendritic cells simultaneously treated with bacterial LPS was carried out. It was stated that phenol fractions completely inhibited the production of TNF-α and the chemokine CXCL8. Polyphenols are well known for their ability to affect macrophage function and, more specifically, the TLR-4 pathway. The authors have submitted a hypothesis regarding the possibility of potential applications of polyphenol–milk protein mixtures in hypoallergenic food products that do not provoke food protein allergies [160]. In a model experiment, the reduction in the allergenicity of bovine β-lactoglobulin (βLG) was determined after its covalent conjugation with (−)-epigallo-catechin 3-gallate (EGCG) and chlorogenic acid (CGA). Western blot analysis and the enzyme-linked immunosorbent assay indicated that the conjugation of βLG with selected polyphenols was effective in reducing the IgE-binding capacity of βLG, which underlined the possibility of reducing βLG allergenic properties through the interaction of proteins and polyphenols [148]. Current studies have demonstrated that enriching milk-fermented products with polyphenol plant extracts (e.g., from chestnut shells, grapeseed, and pomegranate peels) has a beneficial effect on their physicochemical properties and microbiological safety, without reducing the technological characteristics of their production [161].

It was important to undertake studies to evaluate the effects of protein and polypeptide conjugation on changing the allergenicity of the mixture. To this end, a model study was proposed using chlorogenic acid, which is abundant in coffee beans and citrus fruits, and a WPI. It was found that covalent bonding between the components occurred, which could result in the unfolding of the protein structure and the connection of chlorogenic acid molecules. This had a direct effect on lowering the IgE-binding capacity (by about 35%, ELISA) and increasing intestinal digestibility. Chlorogenic acid, through covalent bonds, reacted with the nucleophilic amino acid side chains of proteins, which most likely had the effect of altering the structure of the conformational and linear epitopes. Changes in the rheological characteristics of the resulting mixture were also noted; emulsifying activity, foaming properties, antioxidant capacity of WPI analyzed via DPPH and ABTS radical scavenging, and stability were enhanced [162].

Encouraging results that were obtained using the model method have initiated a series of studies towards designing new, low-allergenic protein–polyphenol products with desirable functional and nutritional characteristics. Polyphenol-enriched milk proteins in the form of fermented beverages, yogurt, and ice cream are the leading products with detailed studies of their rheological and sensory properties, nutritional value, and food safety. Various sources of polyphenolic compounds have been proposed, including fruits, vegetables, spices, and common plants with hitherto underestimated chemical compositions. One such plant is purslane (*Portulaca oleracea*), a common weed that has been found throughout the globe. The extract of its leaves in traditional medicine has been recognized as supporting the treatment of urinary and genital tract ailments and preventing diarrhea; in addition, it is a source of antioxidants, and contains numerous amounts of omega-3 acids, vitamins A and C, and minerals, including calcium, potassium, magnesium, and iron. A study by Osman and colleagues proposed the use of common purslane in doses of 0.4% extract as a source of the bioactive components of phenolic acids, saponins, terpenoids, alkaloids, flavonoids, α-carotene, and α-tocopherol to improve the quality of iced milk. A significant increase (*p* < 0.05) in antioxidant activity was found. The total phenolic and total flavonoid contents also increased in the line of purslane extract concentration. The authors indicated that purslane can be used as a functional food ingredient due to its antioxidant activity [163].

Studies by Sahingil and Hayaloglu have proposed yogurt with 5–20% rosehip (*Rosa canina* L.) pulp. Their sensory analysis pointed out that the yogurt with 20% rosehip pulp had the highest score and acceptability. Together with rosehip addition, an increase in the total phenolic levels was observed. Using the gas chromatographic method, fifty-three volatile compounds were identified, among others, e.g., carboxylic acids, including acetic, butanoic, hexanoic, and octanoic acids. The addition of rosehip pulp increased parameters as water holding capacity, antioxidant capacity, phenolic contents, volatile profile, and sensory attributes of yogurt compared to the control sample [164].

An interesting dietary solution is yogurt with the addition of *Lacticaseibacillus rhamnosus* and aloe vera gel with antioxidant and antimicrobial properties and desirable physicochemical and sensory characteristics. In the realization of the project, different variants of each variable were tested: the concentration of the aloe vera gel (0–5%, *w*/*w*) and storage time (1–28 days). A significant improvement in the viability of *Lacticaseibacillus rhamnosus* to 7.9 cfu/g at shelf life was achieved. Higher antioxidant and antipathogenic activity of the new yogurt, proteolytic content, water retention capacity, and changed sensory characteristics were estimated. The yogurt enriched with *Lacticaseibacillus rhamnosus* and aloe vera had beneficial properties under refrigeration for at least 14 days [165].

Responding to the strategy of utilizing the side streams of the food industry with the extraction of valuable food compounds lost during the primary processing of the raw material is the technology for producing yogurts with peach extracts. Peaches were used as a valuable raw material, containing phenolic acids, anthocyanins, flavonols, and flavanols, as well as vitamins A, B, and C. Microwave-assisted extraction was employed to extract the bioactive compounds. Following this, yogurt was reinforced with peach pectin. This technology led to the formation of a phenol-enriched product with a better texture compared to regularly strained yogurt [166].

Research conducted in the US on the combination of tannic acid with dairy cream has led to the observation of a reduction in the melting rate of ice cream. Tannins were added at 0.75%, 1.5%, and 3%, which caused virtually no change in pH, but an increase in viscosity, leading to gelation at higher tannin levels becoming visible. Microscopic images and particle size distributions confirmed the presence of fat globule clusters in these samples, forming a network at 3%. It was suggested that interactions between the proteins and tannins helped form these clusters [167].

Phenolic compounds are abundant in various species of herbs. Therefore, Kandyliari and co-workers focused on the fortification of dairy products (e.g., kefir, cream cheese, yogurt, and vegan yogurt) with not only aqueous extracts of plant byproducts (e.g., *Citrus aurantium* peel, *Citrus limon* peel, and *Rosa canina* seed) but also herbs (e.g., *Sideritis* spp., *Hypericum perforatum*, *Origanum dictamnus*, *Mentha pulegium* L., *Melissa oficinallis*, *Mentha spicata* L., and *Lavandula angustifolia*). The phytochemical profile of these aqueous extracts determined via mass spectrometry revealed the presence of 162 phytochemicals, of which 128 belonged to the polyphenol family, which includes flavonoids and phenolic acids. The obtained innovative food products possessed high antioxidant and phenolic contents. Sensory estimation pointed to the overall acceptability of new products, but they obtained a score around 3.0–1.0 on a 5-point scale [168].

Products have also been developed with other animal proteins, the allergenicity of which creates health problems. One of the egg proteins, ovalbumin (OVA), was bound with polyphenols as the covalent conjugate OVA–(−)-epigallo-catechin 3-gallate (OVA–EGCG) via a radical or alkaline method. Analysis with LC-MS/MS showed that epigallocatechin-3-gallate was combined with proteins in two sites (Cys74 and Glu347) during the radical reaction and in one site (Cys74) in OVA with the alkaline reaction. FTIR was employed to confirm the formation of a covalent interaction, which led to a higher number of hydroxyl groups introduced on the protein, improving its antioxidant activity. The functional properties of the conjugates were distinct from the protein itself. Digestibility, antioxidant activity, emulsifying properties, and foaming properties increased, but thermal stability reduced. DSC results showed that the structure of OVA was unfolded and epitopes were disrupted, which caused a reduction in the immunoreactivity of strong allergens [169].

The novel approach was the experiment with the allergic C3H/HeJ mouse model. Mice were sensitized to peanut flour; next, they were administered amino acid diets with peanut protein polyphenol aggregates of either low (15%; *w*/*w*) or high (40%; *w*/*w*) complexation ratios of blueberry and cranberry extracts. Mice on diets with high complexation ratios of blueberry and cranberry aggregates showed a significant reduction in peanut-specific plasma IgE. The expression of the basophil surface marker protein CD63 was significantly downregulated. The authors assumed that polyphenolic complexation with peanut protein could prevent the allergenic response of the peanut allergen through the inhibition of IgE [170].

A significant segment of food technology is the development of a directed diet for the elderly. Jolji and her co-workers described bars produced from two protein mixtures: the first included pea protein isolates and rice proteins, and the second one contained pea protein isolates, rice hemp, and sunflower proteins. The sources of polyphenols were banana powder, freeze-dried strawberries, coconut powder, Dutch cacao powder, and vanilla cookies. The highest polyphenol content values were noted for the bars with cacao powder, while the highest flavonoid content was in the bars with cacao powder and banana powder. The addition of the banana powder to the formula caused a higher value of hardness and cutting force. The sensorial analysis revealed that the freeze-dried strawberries and Dutch cacao powder enhanced the flavor of the bars. The authors highlighted the need for further research to deal with special kinds of food for different groups of individuals, e.g., vegans, vegetarians, active lifestyles, and elderly people, to promote health by ensuring the proper level of nutrients [171].

Diaz and co-workers drew attention to the rheological features of bars. They proposed that the bars formulated with whey protein isolate and cranberry freeze-dried particles were softer than their control bars (without cranberry) for up to 31 days of storage, and less elastic for up to 11 days. The protein–polyphenol conjugates increased the nutritional content of the bars and changed their rheological and structural characteristics [172].

It is important to note that protein–polyphenol compounds are new products with higher antioxidant capacity, better functional properties, and low-allergenic potential.

## 7. The Role of Milk Bioactive Peptides on the Organisms

The molecular impact of nutrients and their metabolites on an organism determines the importance of the food for the consumer. The passage of food through the gastrointestinal tract, during which food is transformed into molecules that can be absorbed and used by the body’s cells, depends on nervous and endocrine regulatory mechanisms. Their role is to maintain the proper conditions in the lumen needed for digestion and subsequent absorption. The sensory neurons of the vagus nerve present in the gastrointestinal tract oversee the volume of the stomach and the contents of the intestines. In turn, reactive neural circuits regulate the physiology of digestion. The absorbed nutrients trigger the activation of enteroendocrine cells, which then release intestinal hormones, including serotonin, glucagon-like peptide 1, cholecystokinin, and others, which are needed to initiate food digestion [173]. Due to their different chemical structures and sensitivity to enzymes and bile salts, they undergo hydrolytic processes in the digestive system in different ways [38].

The first, fundamental phase of milk protein digestion occurs in the stomach. This is where the gastric enzymes, mainly pepsin, which is part of the gastric juice, act [174,175]. Casein is digested much more easily compared to whey proteins. In the first step of pepsin digestion, the Phe105-Met106 κ-casein bond is specifically hydrolyzed at pH > 5 [176,177]. Successive coagulation of individual casein fractions occurs via hydrophobic association, accompanied by ionic electrostatic effects, to which whey proteins are not fully susceptible [176,178,179]. Progressive proteolysis in the stomach involves both casein and whey proteins, resulting in the formation of peptides. The process of the coagulation of milk proteins in the stomach affects the dynamics of digestion, which has an impact on the rate of gastric emptying and the passage of food content to the next phases of digestion. Caseins are defined as “slowly” digested proteins that secrete lower but longer-lasting plasma amino acid levels at a later stage, compared to whey proteins, which provide a greater amount of amino acids in a shorter time [177,179,180,181]. Digestion conditions, including the presence of the food matrix, and changes in pepsin concentration and pH values in the stomach have a significant impact on the hydrolysis of casein, milk coagulation, and the properties of the formed coagulates. A study by Yang et al., 2022 [182] indicated a positive correlation between the rate of hydrolysis and the concentration of the enzyme, and the fact that it was pH dependent. In turn, the coagulation time of the protein decreased with increasing pepsin concentration and decreasing pH. The microstructure of the milk coagulum analyzed via confocal laser scanning microscopy revealed that in samples at pH 6.3, 6.0, and 5.7, the network structures and pore sizes were similar to each other and increased at pH 5.3. The rate of casein hydrolysis in the stomach was also affected by prior heat treatment of milk [182]. Ye et al., 2016 [178] using a human gastric simulator (HGS) and prepared simulated gastric fluids (SGFs), showed that heating milk to 37 °C is beneficial, as it facilitates the hydrolysis of casein in the stomach. Casein from heat-treated milk had a loose structure and larger surface area, which facilitated the penetration of the enzyme into the coagulum and accelerated the hydrolysis process, in contrast to non-heat-treated milk, whose casein curds were characterized by a compact structure and small surface area, which make it difficult for pepsin’s diffusion into the coagulum and its hydrolysis [178]. Protein digestion continues in the small intestine, where intestinal proteases and lipases operate [174,175]. Peptidases present in the brush border membrane (BBM) degrade polypeptides and oligopeptides into their monomeric components [183]. Free amino acids are then absorbed by enterocytes [184] and transported to the liver via the portal vein [185].

It is still a major challenge to reduce the allergenicity of dairy products during technological processes and their digestion in the gastrointestinal tract. β-lg is one of the most extensively studied milk proteins in terms of immunoreactivity reduction due to enzymatic hydrolysis under both simulated digestion conditions and enzymatic combinations that have been proposed by various authors. In recent years, it has been shown how many epitopes can be released, and some of them are potent allergenic determinants, e.g., AA: 41–60, 102–124, and 149–162 [186]; AA: 1–8, 25–40, 41–60, 102–124, and 149–162 [187]; and AA: 102–124, 41–60, and 149–162 [188]. Wang with co-authors used alcalase and neutrase in their combined hydrolysis procedure. LC-MS/MS allowed for the detection of peptides with 8–25 amino acid residues, and several allergenic epitopes of β-lactoglobulin (AA: 41–60 and 102–124) were destroyed [189]. The identification of IgE epitopes of β-lactoglobulin (β-LG) after digestion in vitro using human and simulated digestive fluids was analyzed via RP-HPLC-MS/MS. β-LG undertook the digestion process during the gastric phase, in which case no residual β-LG was observed at the end of the duodenal phase. There were overlaps in peptide patterns, where 21 and 18 were duly revealed to be almost similar peptides in gastric and duodenal digestion. Digestion products related to β-LG corresponded to five high-frequency IgE-binding synthetic peptides, namely 43–60, 47–62, 86–99, 86–100, and 135–147, which were released upon in vitro digestion, particularly with a simulated fluid [190].

Bioactive peptides are released as a result of lactoferrin digestion in the acidic environment of gastric juice, and present pepsin. Among these peptides, two were found to be stable (lactoferricin and lactoferampin) and possessed strong antimicrobial properties. Lactoferricin is a cationic peptide, with its sequence (FKCRRWQWRMKKLGAPSITCVRRAF) corresponding to the fragments 17–41 of lactoferrin. Lactoferricin possesses an effect against Gram-positive and Gram-negative bacteria and fungi, parasites, viruses, and tumor cells. [191,192]. Lactoferrampin is also a cationic peptide (WKLLSKAQEKFGKNKSR), including the residues from 268 to 284 of the N1 domain of lactoferrin. Its antibacterial and antifungal properties have been described [193].

During the development of an infant organism, breastfeeding and what comes with it—the ability to pass on valuable peptides—play a significant role at the beginning of life. Peptides that are absorbed while passing through the gastrointestinal system can reach the bloodstream and exert bioactivity. The in vitro study with the use of human intestinal Caco-2 cell monolayers found that the number of absorbed peptides depended on different concentrations: 44 peptides out of 169 peptides were detected at 10 μg/mL; 124 peptides out of 204 peptides were detected at 100 μg/mL; and 175 peptides out of 236 peptides was detected at 1000 μg/mL. Four peptides that were released from β-casein (NLHLPLP, PLAPVHNPI, PLMQQVPQPIPQ, and FDPQIPK) crossed to the basolateral chamber of the Caco-2 cell monolayers at all three concentrations. Three peptides were characterized as known angiotensin-converting enzyme (ACE) inhibitors; one peptide was antimicrobial, and one possessed antioxidant activity [194]. The presence of milk protein peptides in the intestinal lumen can have a stimulating effect on mucus production or mineral absorption or promote the inhibition of inflammation [77]. Peptides formed via enzymatic digestion exhibit health-promoting properties, among which we can also include antihypertensive, anticancer, antioxidant, antidiabetic, antimicrobial, and many other properties [195,196,197]. For this reason, bioactive milk peptides have found their use as dietary supplements, functional food ingredients, or pharmaceuticals to improve health and thereby reduce the incidence of chronic diseases [82]. For example, peptides that inhibit ACEs may be used to treat hypertension. In vitro studies conducted by Ibrahim et al., 2017 [198] showed that both caseins and whey proteins, found in goat milk, released peptides with this effect upon their exposure to gastric pepsin [198]. Identical properties were found for an αs2-casein fragment with residues 25–41, formed from a hydrolysate of bovine casein treated with trypsin [199]. On the other hand, peptides that inhibit the activity of dipeptidyl peptidase IV (DPP-IV) may present a strategy for treating type 2 diabetes (T2D). The inhibitory ability of DPP-IV has been detected in peptides released from bovine whey protein concentrates, more specifically α-lactalbumin, treated with trypsin [200]. Taghipour et al., 2023 [197] found that antimicrobial properties could be detected in camel milk proteins. Peptides released from casein treated with trypsin proved to be the most effective against *Klebsiella pneumoniae*, and in the case of *Pseudomonas aeruginosa*, treated with pepsin and trypsin in turn. Whey protein hydrolysates formed by pepsin exhibited their strongest antibacterial activity against *Staphylococcus aureus* and *Escherichia coli*, of which in the case of the last group of bacteria, an identical effect was obtained using trypsin separately [197].

It is important to note that dairy milk products passed through the gastrointestinal tract release different bioactive peptides characterized as antihypertensive, anticancer, antioxidant, antidiabetic, and antimicrobial compounds.

## 8. The Effects of Bioactive Polyphenols and Their Metabolites in Organisms during their Passage through the Gastrointestinal Tract

The bioaccessibility and bioavailability of polyphenols in organisms from plant material have been correlated with their concentration in raw material. They are metabolized in the digestion tract, which depends on the dietary food matrix, the activity of the gut microbiota, and the state of the organism (regular or pathological).

The first stage of polyphenol digestion takes place in the mouth, where food is broken down (mastication/crushed) and comes into contact with saliva. Mechanical grinding of food will ensure greater bioavailability and interaction of polyphenols released from food with digestive enzymes at later stages. The structure of the food can affect the outcome of polyphenol mastication, which, in turn, affects their further absorption in the digestive tract [201,202]. From the esophagus, polyphenols make their way to the stomach, where they can be released from the food matrix and hydrolyzed under an acidic environment [203,204]. Generally, phenolic compounds are not absorbed in the stomach. An exception may be phenolic acids, which, as small phenolic compounds, are not strongly bound to the food matrix, allowing them to be absorbed as early as the digestion stage in the stomach. However, they can promote the secretion of enteric hormones, including glucagon-like peptide 1 (GLP-1), which is crucial for the regulation of blood glucose concentration [205,206,207]. Chyme from the stomach is transported to the duodenum. The small intestine is the target site for the absorption and transformation of phenolic compounds. The pH of the digestive environment is changed, from acidic to alkaline, as a result of pancreatic juices and bile, and because of that phenolic compounds become unstable [208,209]. The absorption of polyphenol degradation products from the small intestine into enterocytes is determined by a number of dependent factors, among which we can mention the chemical structure, hydrophobicity, glycosidic moiety, or polymerization of the compounds [210]. Phenolic compounds in the form of glycosidic conjugates are hydrolyzed through the actions of lactase phlorizin hydrolase (LPH) [211,212]. Following enzymatic hydrolysis, in which lipophilic aglycones are released, the route of passage into epithelial cells is passive diffusion. In the case of glucosides more polar than aglycone, an alternative is their active transport across the cell membrane into enterocytes, dependent on the sodium glucose transporter (SGLT-1), where they are sequentially hydrolyzed by cytosolic β-glucosidase (CBG) [202,213,214], allowing them to eliminate glycosidic groups. It has been found that anthocyanins inhibit gastrointestinal lumen enzymes that are involved in lipid and carbohydrate absorption, maintain the integrity of the intestinal barrier and prevent endotoxemia, and modulate the metabolism of hormones produced via the gastrointestinal tract [215].

Polyphenols that enter the bloodstream usually undergo a second phase of biotransformation in the liver. If the polyphenol glycosides are not absorbed at this stage, it means that they are not substrates for the aforementioned enzymes and will be degraded in the large intestine [216,217]. The microbiota in the large intestine enables the metabolization of polyphenols that end up in the colon [25]. The process of the bioconversion of phenolic compounds depends on the quality of the microbiota, which varies depending on the individual, the structure of the polyphenols, and the food matrix from which they are coming [218]. Polyphenols are degraded to low molecular weight compounds, which elevates their bioavailability and allows for easier absorption [202]. Due to the presence of colonic bacterial enzymes, deconjugation, dehydroxylation, and ring fission are possible, resulting in the formation of (mainly) phenolic acids [219,220], which can then enter the circulatory system or actively affect the functions of cells in the colon, positively modulating the intestinal microbiota. Conjugated polyphenols are not absorbed in the small intestine and pass to the colon, where the microbiota transforms these polyphenols into absorbable products. Predominant groups (i.e., phenolic acids with 0–3 aromatic hydroxyls, or their mono- or di-methoxy analogues) originating from varying substrates (i.e., chlorogenic acids, flavanols, proanthocyanidins, and aflavins) are produced.

The study on the stability and absorption in a simulated gut digestion condition is usually conducted with cell lines, e.g., blueberry anthocyanins were carried on with the use of the blueberry malvidins transport through Caco-2 cells. The malvidins are characterized by their relatively higher absorption efficiency percentages greater hydrophobic properties compared to other anthocyanins [221].

Valuable scientific findings relate to studies in which the authors compared the properties of the raw material and the product obtained from it. Lingua (2018) and co-workers evaluated the effect of simulated gastrointestinal digestion on the phenolic profile and the antioxidant capacity of grapes and red wines. They observed that the bioaccesibility of polyphenols from grapes and wines was similar. In grapes, polyphenols were released during mouth and stomach digestion. Wine polyphenols were already bioaccesible, probably due to earlier technological processes. Less than 52% of polyphenols would be potentially bioaccessible, and anthocyanins were the most resistant polyphenols to digestion [201].

It is important to note that digestion is characterized by the presence of a second phase of biotransformation in the liver and is conjugated due to microbiota polyphenols are not absorbed in the small intestine but are passed to the gut for further hydrolysis.

## 9. Docking: Potential Impact Mechanism of the Protein–Polyphenol Complex Reaction on Organisms

In silico methods are developing extremely rapidly. Thanks to bioinformatics, analyses of arbitrary configurations between different food components, mainly bioactive ones, are possible. This makes it possible to predict, to a very close approximation, the mechanisms of their formation, changes in structure and properties, and the consequences of their effects on the organism. Combining food ingredients from different sources (animal and plant) is a complex process with different consequences for the structure and properties of the new products.

The molecular interaction of cow’s milk proteins (e.g., α-lactalbumin, β-lactoglobulin, αs1-casein, and β-casein) with cyanidin-3-*O*-glucoside was determined using computational methods via docking analysis. The results showed that the complexes were stabilized by hydrogen and hydrophobic interactions. The radius of gyration, number of hydrogen bonds, radial distribution function, and interaction energy showed that αs1-casein and β-casein had more hydrophobic and hydrogen-bonding sites with cyanidin-3-O-glucoside than whey proteins, and β-casein was described as the best carrier among the four analyzed milk proteins. Protein carriers can improve the short-term bioavailability and stability of anthocyanins, which is important in regulating human health [222].

Three different polyphenols (eriodictyol, luteolin, and apigenin, from PubChem) were complexed with β-lactoglobulin from the Protein Data Bank. The authors employed AutoDock 4.2 software for their molecular docking calculations and quantum mechanics/molecular mechanics (QM/MM) studies. For eriodictyol and luteolin, van der Waals, hydrophobic, and hydrogen bonding interactions were the main interacting forces, whereas for apigenin, hydrophobic and van der Waals interactions were detected. Apigenin and luteolin act had better antioxidant properties than eriodictyol. However, eriodictyol forms a more stable complex with β-lactoglobulin than the other two polyphenols. Presumably, it can be thought that eriodictyol may have a higher affinity for this protein. The authors pointed that apigenin and luteolin were the more active antioxidants than eriodictyol in the protein environment, probably due to their conjugation, which stabilized the radicals formed by the removal of one H atom from the polyphenols. The interaction of eriodictyol with β-lactoglobulin reduced the antioxidant activity of eriodictyol inside the protein environment [223].

In terms of the docking studies, Li and co-workers (2020) carried out the conjugation of βLG with 3,4-dihydroxybenzoic acid, gallic acid, syringic acid, caffeic acid, ferulic acid, and chlorogenic acid. They found that cinnamic acid derivatives possess a stronger binding affinity than benzoic acid derivatives along the line caffeic acid > chlorogenic acid > ferulic acid > syringic acid > 3,4-dihydroxybenzoic acid > gallic acid. The process mainly involved hydrophobic reactions. The authors emphasized the importance of these observations in terms of the use of protein–polyphenol conjugates in both the food and pharmaceutical industries [224].

The current knowledge, literature data, and predictive values of docking analyses allow us to assume a potential mechanism of defense of the allergic body against allergic milk proteins.

**Figure 3:**
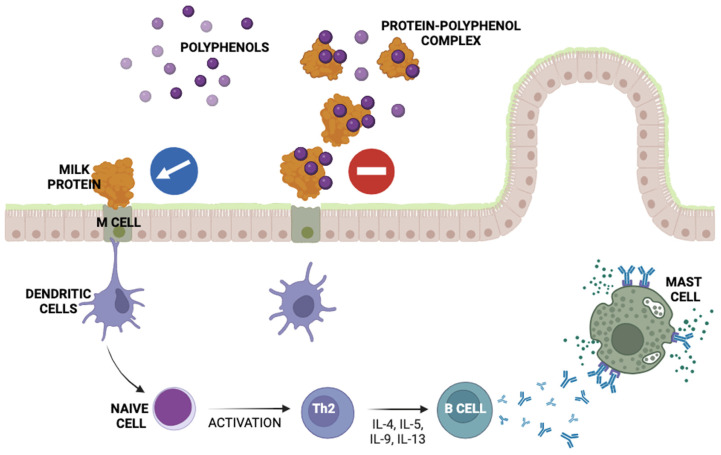
Absorption of milk proteins (allergens) and protein–polyphenol complexes through the intestinal wall.

Specific IgE antibodies to milk epitopes produced during the primary sensitization phase will not find binding sites in protein–polypeptide complexes. This may be a consequence of the occupation of these attachment sites by polyphenol metabolites, and thus the proteins in the complexes should be protected against easy penetration through the intestinal barrier. These complexes should not develop an allergic reaction (Figure 3).

The docking analysis allows for determining the inhibitory effect of conjugates of epigallocatechin-3-O-gallate and α-glucosidase. Together with its hypoglycemic effect via the PI3K/AKT signaling pathway, conjugates’ antidiabetic properties were shown. The binding site between α-glucosidase and EGCG was identified as a form of stable complex, an active site pocket of α-glucosidase with five intermolecular hydrogen bonds [225].

The results of in silico analyses and majority docking analyses present possible mechanisms of phenol–protein complex formation, their physiological action, and their activities. Their theoretical results provide the background for further studies on cellular models, animal models, and eventually volunteers.

An example of this can be found in allergenicity studies, where computer databases hold a huge number of potential epitopes. But when translating this knowledge into specific allergy cases among patients, it will be important what products, not epitopes, they have to avoid. On the other hand, the help of bioinformatics is indispensable for developing new food products. In this way, it is possible to provide a personalized diet for vulnerable groups, where a targeted diet is needed.

It is important to note that docking allows us to predict the possible mechanism of activity of the protein–polyphenol complexes in organisms.

## 10. Conclusions

This review presents scientific findings on the bioactive components of dairy milk materials and plant polyphenols, their hydrolysates, and conjugates. Apart from their nutritional, structural, and functional properties, milk proteins are characterized by the presence of strong allergens (e.g., β-lg, α-la, αs1-casein, and κ-casein) and peptides occurring during the hydrolysis of milk during their technological processes and digestion. The selected bacterial strains reduce the immunoreactivity of fermented milk products (e.g., kefir, yogurt, buttermilk, whey drinks, and colostrum). It was pointed out that the milk of other animals (e.g., mare, goat, camel, and donkey) is also a source of bioactive peptides with anti-inflammatory, antiaging, and antimicrobial features that enhance immunoactivity. Polyphenols isolated from plant sources (e.g., fruits, vegetables, herbs, spices, etc.) are valuable components with antioxidant properties, improving the rheological, organoleptic, and prebiotic properties of polyphenols.

The conscious combination of proteins and polyphenols can also effectively reduce the allergenicity of new products, which is extremely important when evaluating a marketed product. Assessing the occurring interactions in protein–polyphenol conjugates and predicting new chemical structures and properties is within the scope of in silico analyses, including molecular docking techniques. This form of analysis helps to clarify and understand the changes that may occur as a result of the conjugation and formation of the protein–polyphenol chemical structure. At the same time, the immune properties that such a conjugate will have can be estimated.

The enzymatic digestion of the proteins that have been described in this review illustrates the wealth of peptides with different characteristics that appear in the digestive system during hydrolysis in its different sections. Digestion of polypeptide compounds is more complicated, as they undergo a second phase of biotransformation in the liver. Model studies on polyphenol–protein conjugates are being performed on cell lines (Caco2) and laboratory animals, but the challenge still facing scientists is to demonstrate the effect of polyphenol–protein adducts on the host immune system under physiological and pathological states (mainly allergies and various gastrointestinal diseases). This will form the basis for providing an appropriate diet for consumers in vulnerable groups, and further research involving volunteers is needed for this purpose. Currently, there are not enough research data to authoritatively answer the question presented in the title regarding bioactive-fermented dairy products and phenolic compounds that can be consumed together or separately. However, the data that have been presented in the literature have proven that satisfactory results in reducing allergenicity have already been achieved.

In conclusion, it is worth emphasizing that in silico prediction and scientific reports to date indicate the validity of combining proteins with polyphenols.

## Figures and Tables

**Table 1:** Properties of bioactive milk products.

Source	Properties	Reference
**MILK**		
Raw cow’s milk	- Prescence of vitamins A and D and immunomodulatory cytokines (TGF-β2, osteopontin, and IL10 (low levels)) - Immunomodulatory cytokines create the environment, which promotes regulatory T cell production (developing and maintaining oral tolerance in the gut with IgA and IgG4)	[94,95]
Mare’s milk	- Inhibiting the growth of pathogenic bacteria - Reduction in IgE levels - Increase in the number of Treg cells (immunosuppressive role) - Decrease in IL-4 expression and increase in TL-4 expression (positive effect on the innate response)	[73,75]
**Fermented Milk Beverages**		
Camel milk	- Antioxidant peptides	[90]
Donkey milk	- *Staphylococcus aureus* growth reduction - Inhibition of HSV-1 replication	[91]
Sheep milk	- Antioxidant peptides - Antimicrobial peptides against *Enterococcus faecalis*, *Salmonella typhimurium*, *Bacillus cereus*, and *Escherichia coli* - Reduction in the excess of pro-inflammatory cytokines, namely TNF-α, IL-6, and IL-1β	[89]
Yogurt	- Increased levels of IFN-γ, IgG1, and IL-10 against viral infection - Decrease in inflammatory cytokines, namely TNF-α and IL-6 - Peptides with ACE-inhibiting, antimicrobial, and DPP-IV-inhibiting properties - Induction of immune tolerance in CMA	[49,50]
Kefir	- Protective functions against metabolic syndrome, obesity, hypercholesterolemia, and immunostimulating effects - Hypotensive, hypolipidemic, and hypocholesterolemic effects - Reduction in the allergenicity of β-lactoglobulin (main milk allergen)	[51,54]
Whey	- Prevention of enteritis and clinical symptoms of food allergy - Bioactive whey proteins - Stimulatory effect on splenocytes and lymphocytes - Phagocytic, granulocyte, and NK cell activity - Promote the differentiation of T- and B-lymphocytes - Increase CD8+ and CD4+ cells - Inhibition of cytokines, namely TNF-α, IL-1 α, and IL-6	[63,96]
Buttermilk	- Increased levels of TNF-α and IFN-γ - Reduction in the mean values of plasma lipid levels	[70]
Kumis	- Well tolerated by people with cow’s milk intolerance, atopic dermatitis, and chronic gastrointestinal diseases	[77]
Colostrum	- Antimicrobial activity of bioactive peptides against *Escherichia coli*—ingredient of functional food against diarrhea - Increase in NK cell activity	[16,97]

**Table 2:** Prebiotic properties of polyphenols.

Source	Properties	Type of Research	Reference
**Anthocyanins**			
Black rice	*Bifidobacterium bifidum* ↑ *Bifidobacterium adolescentis* ↑ *Bifidobacterium infantis* ↑ *Lactobacillus acidophilus* ↑	In vitro	[131]
Purple sweet potato	*Bifidobacterium* spp. ↑ *Lactobacillus/Enterococcus* spp.↑ *Bacteroides-Prevotella* ↓ *Clostridium histolyticum* ↓	In vitro	[132]
Dark sweet cherry powder	*Akkermansia* ↑	In vivo	[133]
Cranberry extract	*Akkermansia* spp. ↑ Reduced triglycerides ↓ Reduced oxidative stress ↓ Reduced intestinal inflammation ↓	In vivo	[134]
**Flavonols**			
Beverage with a high content of cocoa flavonols	*Lactobacillus* spp. ↑ *Bifidobacterium* spp. ↑	Human study	[135]
Cocoa extract	*Lactobacillus* spp. ↑ *Bifidobacterium* spp. ↑	In vitro	[135]
**Other Polyphenols**			
Blueberry extract *	Inhibited weight gain Restored normal lipid metabolism Modulation of the composition of the intestinal microbiota	In vivo	[136]
Red and white grapes *	*Lactobacillus* spp. ↑ *Bifidobacterium* spp. ↑	In vitro	[137]
Non-alcoholic extract of red wine **	*Lactobacillus* spp. ↑ *Bifidobacterium* spp. ↑ *Clostridium* spp. ↓ *Propionibacterium* spp. ↓ *Bacteroides* spp. ↓	In vivo	[138]

* Total phenolic compounds; ** anthocyanins, flavonols, and phenolic acids, along with complex phenols and tannins with various degrees of polymerization. ↑ increase in bacterial count/characteristics, ↓ decrease in bacterial count/characteristics.

## Data Availability

Not applicable.
